# Supplementary material for: Analysis of 567,758 randomized controlled trials published over 30 years reveals trends in phrases used to discuss results that do not reach statistical significance
Source: PLoS Biol. 2022 Feb 18;20(2):e3001562. doi: 10.1371/journal.pbio.3001562 (PMC8893613; doi:10.1371/journal.pbio.3001562)
Supplement: S1 Fig — Prevalence estimates are shown as dots, together with the linear regression model fit and 95% CI. The data can be found here: https://github.com/wmotte/almost_significant/tree/main/Fig_and_Data. BF, Bayes factor; CI, confidence interval. (DOCX) [file pbio.3001562.s001.docx]

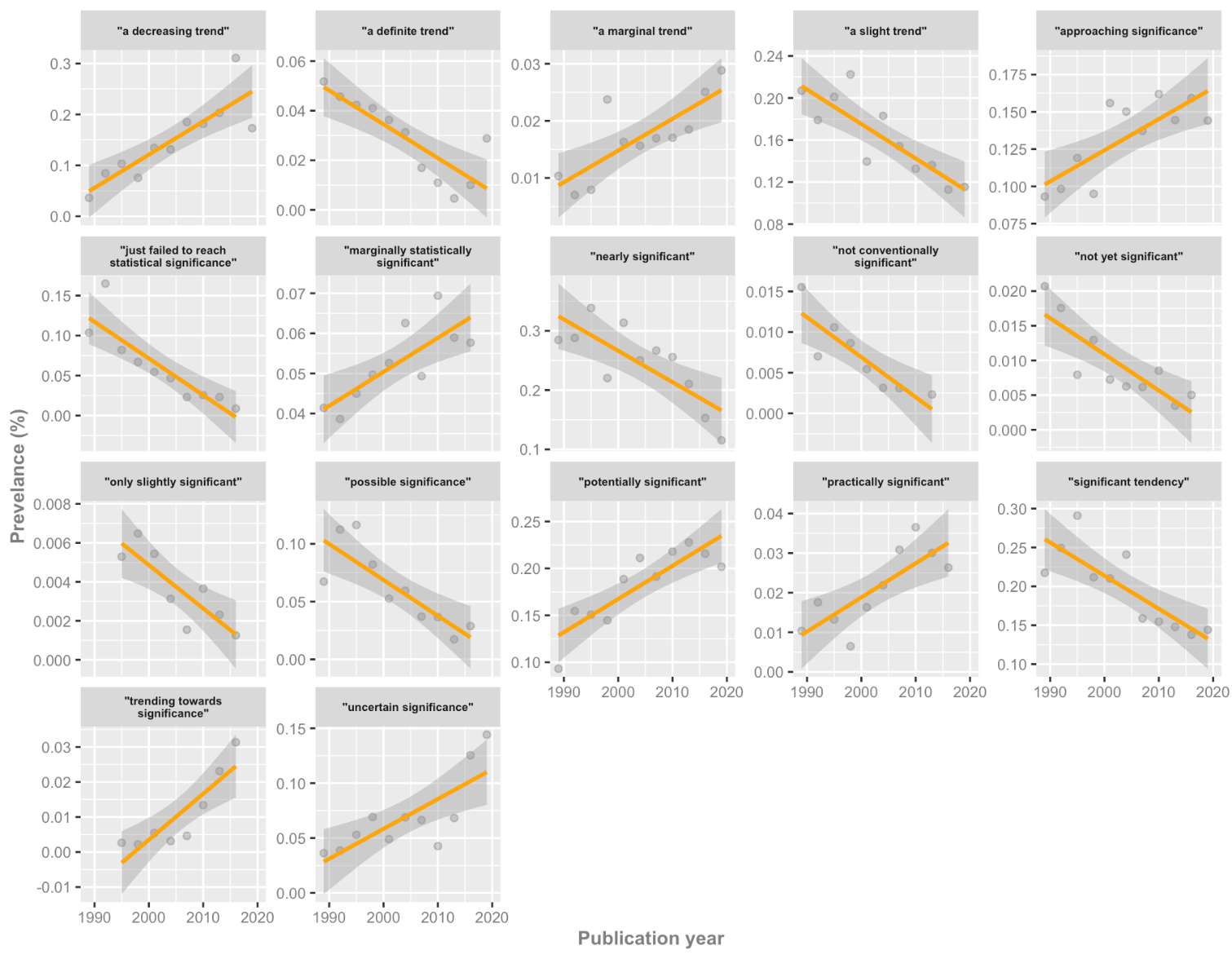


**S1 Fig**. Temporal plots for phrases with ‘strong’ evidence (i.e., Bayes factors between 10–100) for temporal change. Prevalence estimates are shown as dots, together with the linear regression model fit and 95% confidence interval. The data can be found here: <https://github.com/wmotte/almost_significant/tree/main/Fig_and_Data>
