# Supplementary material for: Analysis of 567,758 randomized controlled trials published over 30 years reveals trends in phrases used to discuss results that do not reach statistical significance
Source: PLoS Biol. 2022 Feb 18;20(2):e3001562. doi: 10.1371/journal.pbio.3001562 (PMC8893613; doi:10.1371/journal.pbio.3001562)
Supplement: S2 Fig — Error bars represent the proportional 95% CI. The associated median P value is presented in the upper left corner of each phrase. CI, confidence interval. (DOCX) [file pbio.3001562.s002.docx]

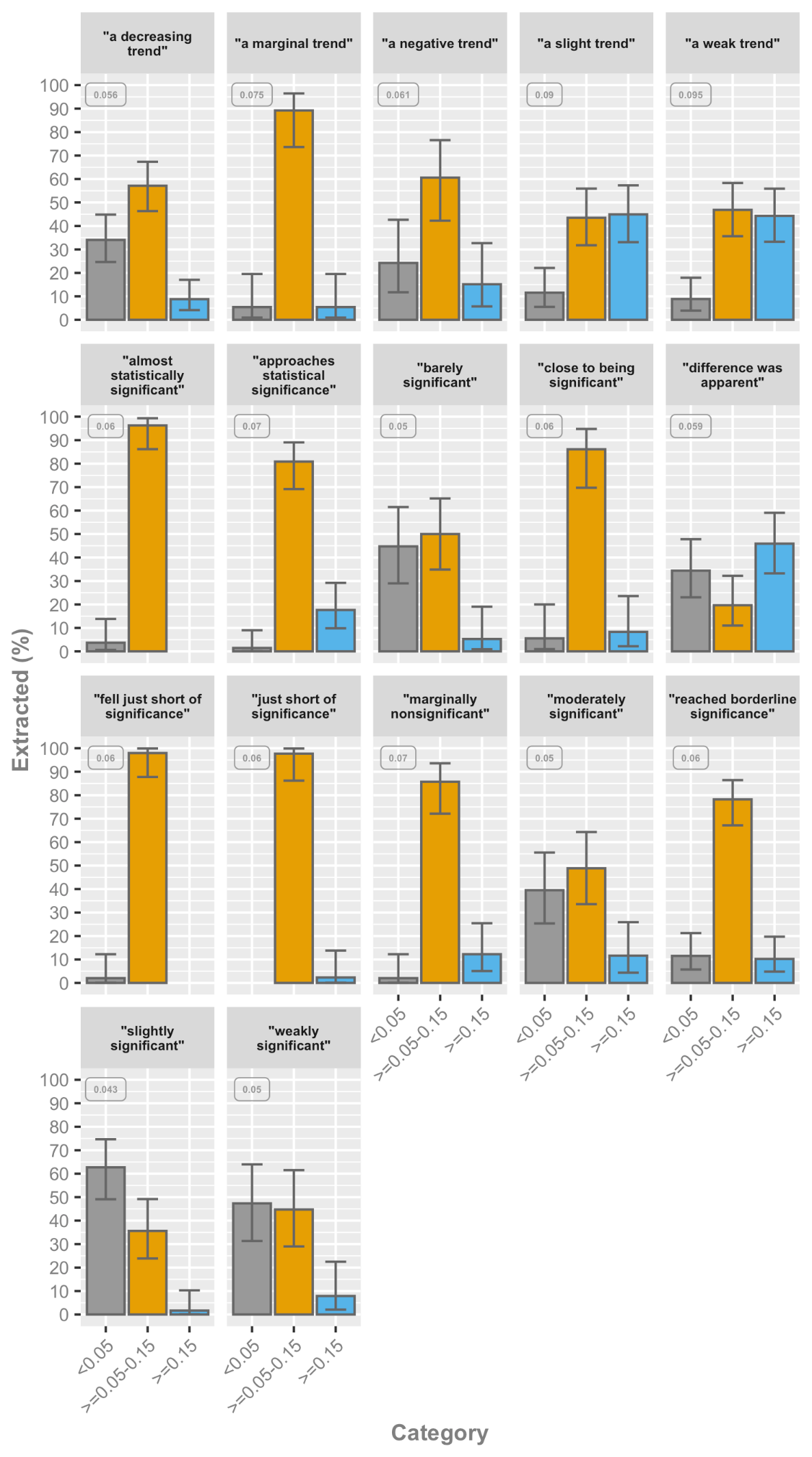


**S2 Fig**. Category percentages for the phrases describing non-significant results with the number of manually extracted P values with occurrences between 30 and 100 times in our manual analysis. Error bars represent the proportional 95% confidence interval. The associated median P value is presented in the upper left corner of each phrase
