# Supplementary material for: Analysis of 567,758 randomized controlled trials published over 30 years reveals trends in phrases used to discuss results that do not reach statistical significance
Source: PLoS Biol. 2022 Feb 18;20(2):e3001562. doi: 10.1371/journal.pbio.3001562 (PMC8893613; doi:10.1371/journal.pbio.3001562)
Supplement: S1 Table — (DOCX) [file pbio.3001562.s003.docx]

**S1 Table**. The 505 pre-defined phrases associated with reporting non-significant results.

|  | **1** | **2** | **3** | **4** | **5** |
| --- | --- | --- | --- | --- | --- |
| **1** | barely not statistically significant | approaching an acceptable significance level | fell narrowly short of significance | nearly significant tendency | practically significant |
| **2** | a barely detectable statistically significant difference | approaching borderline significance | fell only marginally short of significance | nearly, but not quite significant | probably not experimentally significant |
| **3** | a borderline significant trend | approaching borderline statistical significance | fell only short of significance | near-marginal significance | probably not significant |
| **4** | a certain trend toward significance | approaching but not reaching significance | fell short of significance | near-significant | probably not statistically significant |
| **5** | a clear tendency to significance | approaching clinical significance | fell slightly short of significance | near-to-significance | probably significant |
| **6** | a clear trend | approaching close to significance | fell somewhat short of significance | near-trend significance | provisionally significant |
| **7** | a clear, strong trend | approaching conventional significance levels | felt short of significance | nominally significant | quasi-significant |
| **8** | a considerable trend toward significance | approaching conventional statistical significance | flirting with conventional levels of significance | non-insignificant result | questionably significant |
| **9** | a decreasing trend | approaching formal significance | heading towards significance | non-significant in the statistical sense | quite close to significance at the 10% level |
| **10** | a definite trend | approaching independent prognostic significance | hint of significance | not absolutely significant but very probably so | quite significant |
| **11** | a distinct trend toward significance | approaching marginal levels of significance | hovered around signiﬁcance | not as significant | rather marginal significance |
| **12** | a favorable trend | approaching marginal significance | hovered at nearly a significant level | not clearly significant | reached borderline significance |
| **13** | a favourable statistical trend | approaching more closely significance | hovering closer to statistical significance | not completely significant | reached near significance |
| **14** | a little significant | approaching our preset significance level | hovers on the brink of significance | not completely statistically signiﬁcant | reasonably significant |
| **15** | a margin at the edge of significance | approaching prognostic significance | in the edge of significance | not conventionally significant | remarkably close to significance |
| **16** | a marginal trend | approaching significance | in the verge of significance | not currently significant | resides on the edge of significance |
| **17** | a marginal trend toward significance | approaching the traditional significance level | inconclusively significant | not decisively significant | roughly significant |
| **18** | a marked trend | approaching to statistical significance | indeterminate significance | not entirely significant | scarcely significant |
| **19** | a mild trend | approaching, although not reaching, significance | indicative significance | not especially significant | significant at the .07 level |
| **20** | a moderate trend toward significance | approaching, but not reaching, significance | is just outside the conventional levels of significance | not exactly significant | significant tendency |
| **21** | a near-significant trend | approximately significant | just about significant | not extremely significant | significant to some degree |
| **22** | a negative trend | approximating significance | just above the arbitrary level of signiﬁcance | not formally significant | significant, or close to significant effects |
| **23** | a nonsignificant trend | arguably significant | just above the margin of significance | not fully significant | significantly significant |
| **24** | a nonsignificant trend toward significance | as good as significant | just at the conventional level of significance | not globally significant | similar but not nonsigniﬁcant trends |
| **25** | a notable trend | at the brink of significance | just barely below the level of significance | not highly significant | slight evidence of significance |
| **26** | a numerical increasing trend | at the cusp of significance | just barely failed to reach significance | not markedly significant | slight non-significance |
| **27** | a numerical trend | at the edge of significance | just barely insignificant | not moderately significant | slight significance |
| **28** | a positive trend | at the limit of significance | just barely statistically signiﬁcant | not non-significant | slight tendency toward significance |
| **29** | a possible trend | at the limits of significance | just beyond significance | not numerically significant | slightly above the level of signiﬁcance |
| **30** | a possible trend toward significance | at the margin of significance | just borderline significant | not obviously signiﬁcant | slightly below the level of signiﬁcance |
| **31** | a pronounced trend | at the margin of statistical significance | just escaped significance | not overly significant | slightly exceeded signiﬁcance level |
| **32** | a reliable trend | at the verge of significance | just failed significance | not quite borderline significance | slightly failed to reach statistical signiﬁcance |
| **33** | a robust trend toward significance | at the very edge of significance | just failed to be significant | not quite reach the level of significance | slightly insignificant |
| **34** | a significant trend | barely below the level of significance | just failed to reach statistical significance | not quite significant | slightly less than needed for significance |
| **35** | a slight slide towards significance | barely escaped statistical significance | just failing to reach statistical significance | not quite within the conventional bounds of statistical significance | slightly marginally significant |
| **36** | a slight tendency toward significance | barely escapes being statistically significant at the 5% risk level | just fails to reach conventional levels of statistical significance | not reliably signiﬁcant | slightly missed being of statistical significance |
| **37** | a slight trend | barely failed to attain statistical significance | just lacked significance | not remarkably signiﬁcant | slightly missed statistical significance |
| **38** | a slight trend toward significance | barely fails to attain statistical significance at conventional levels | just marginally significant | not significant by common standards | slightly missed the conventional level of significance |
| **39** | a slightly increasing trend | barely insignificant | just missed being statistically significant | not significant by conventional standards | slightly missed the level of statistical significance |
| **40** | a small trend | barely missed statistical significance | just missing significance | not significant by traditional standards | slightly missed the margin of significance |
| **41** | a statistical trend | barely missed the commonly acceptable significance level | just on the verge of significance | not significant in the formal statistical sense | slightly not significant |
| **42** | a statistical trend toward significance | barely outside the range of significance | just outside accepted levels of significance | not significant in the narrow sense of the word | slightly outside conventional statistical significance |
| **43** | a strong tendency towards statistical significance | barely significant | just outside levels of significance | not significant in the normally accepted statistical sense | slightly outside the margins of significance |
| **44** | a strong trend | below but verging on the statistical significant level | just outside the bounds of significance | not significantly significant but clinically meaningful | slightly outside the range of significance |
| **45** | a strong trend toward significance | better trends of improvement | just outside the conventional levels of significance | not statistically quite significant | slightly outside the significance level |
| **46** | a substantial trend toward significance | bordered on a statistically significant value | just outside the level of significance | not strictly significant | slightly outside the statistical significance level |
| **47** | a suggestive trend | bordered on being significant | just outside the limits of significance | not strictly speaking significant | slightly significant |
| **48** | a trend close to significance | bordered on being statistically significant | just outside the traditional bounds of significance | not technically significant | somewhat marginally significant |
| **49** | a trend significance level | bordered on but was not less than the accepted level of significance | just over the limits of statistical significance | not that significant | somewhat short of significance |
| **50** | a trend that approached significance | bordered on significant | just short of significance | not to an extent that was fully statistically signiﬁcant | somewhat significant |
| **51** | a very slight trend toward significance | borderline conventional significance | just shy of significance | not too distant from statistical significance at the 10% level | somewhat statistically significant |
| **52** | a weak trend | borderline level of statistical significance | just skirting the boundary of significance | not too far from significant at the 10% level | strong trend toward significance |
| **53** | a weak trend toward significance | borderline signiﬁcant | just tendentially signiﬁcant | not totally significant | sufficiently close to significance |
| **54** | a worrying trend | borderline significant trends | just tottering on the brink of significance at the 0.05 level | not unequivocally significant | suggestive but not quite significant |
| **55** | all but significant | close to a marginally significant level | just very slightly missed the significance level | not very definitely significant | suggestive of a significant trend |
| **56** | almost achieved significance | close to being significant | leaning towards significance | not very definitely significant from the statistical point of view | suggestive of statistical significance |
| **57** | almost approached significance | close to being statistically signiﬁcant | leaning towards statistical significance | not very far from significance | suggestively significant |
| **58** | almost attained significance | close to borderline signiﬁcance | likely to be significant | not very significant | tailed to insignificance |
| **59** | almost became significant | close to the boundary of significance | loosely significant | not very statistically significant | tantalisingly close to significance |
| **60** | almost but not quite significant | close to the level of significance | marginal significance | not wholly significant | technically not significant |
| **61** | almost clinically significant | close to the limit of significance | marginally and negatively significant | not yet significant | teetering on the brink of significance |
| **62** | almost insignificant | close to the margin of significance | marginally insignificant | not strongly significant | tend to significant |
| **63** | almost marginally significant | close to the margin of statistical significance | marginally nonsignificant | noticeably signiﬁcant | tended to approach significance |
| **64** | almost non-significant | closely approaches the brink of signiﬁcance | marginally outside the level of significance | on the border of significance | tended to be significant |
| **65** | almost reached statistical significance | closely approaches the statistical significance | marginally significant | on the borderline of significance | tended toward significance |
| **66** | almost significant | closely approximating significance | marginally significant tendency | on the borderlines of significance | tendency toward significance |
| **67** | almost significant tendency | closely not significant | marginally statistically significant | on the boundaries of signiﬁcance | tendency toward statistical significance |
| **68** | almost statistically significant | closely significant | may not be signiﬁcant | on the boundary of signiﬁcance | tends to approach signiﬁcance |
| **69** | an adverse trend | close-to-signiﬁcant | medium level of significance | on the brink of significance | tentatively signiﬁcant |
| **70** | an apparent trend | did not achieve conventional threshold levels of statistical significance | mildly signiﬁcant | on the cusp of conventional statistical significance | too far from signiﬁcance |
| **71** | an associative trend | did not exceed the conventional level of statistical significance | missed narrowly statistical significance | on the cusp of significance | trend bordering on statistical significance |
| **72** | an elevated trend | did not quite achieve acceptable levels of statistical significance | moderately significant | on the edge of significance | trend in a significant direction |
| **73** | an encouraging trend | did not quite achieve significance | modestly significant | on the limit to significant | trend in the direction of significance |
| **74** | an established trend | did not quite achieve the conventional levels of significance | narrowly avoided significance | on the margin of significance | trend significance level |
| **75** | an evident trend | did not quite achieve the threshold for statistical significance | narrowly eluded statistical significance | on the threshold of significance | trending towards significance |
| **76** | an expected trend | did not quite attain conventional levels of significance | narrowly escaped significance | on the verge of significance | trending towards significant |
| **77** | an important trend | did not quite reach a statistically significant level | narrowly evaded statistical significance | on the very borderline of significance | uncertain significance |
| **78** | an increasing trend | did not quite reach conventional levels of statistical significance | narrowly failed significance | on the very fringes of signiﬁcance | vaguely significant |
| **79** | an interesting trend | did not quite reach statistical significance | narrowly missed achieving significance | on the very limits of significance | verged on being significant |
| **80** | an inverse trend toward signiﬁcance | did not reach the traditional level of signiﬁcance | narrowly missed overall significance | only a little short of significance | verging on significance |
| **81** | an observed trend | did not reach the usually accepted level of clinical significance | narrowly missed significance | only just failed to meet statistical significance | verging on the statistically significant |
| **82** | an obvious trend | difference was apparent | narrowly missed standard significance levels | only just insignificant | verging-on-significant |
| **83** | an overall trend | direction heading towards significance | narrowly missed the significance level | only just missed significance at the 5% level | very close to approaching significance |
| **84** | an unexpected trend | does not appear to be sufficiently significant | narrowly missing conventional significance | only marginally fails to be significant at the 95% level | very close to significant |
| **85** | an unexplained trend | does not narrowly reach statistical significance | near limit significance | only marginally nearly insignificant | very close to the conventional level of significance |
| **86** | an unfavorable trend | does not reach the conventional significance level | near miss of statistical significance | only marginally significant | very close to the cut-off for significance |
| **87** | appeared to be marginally significant | effectively significant | near nominal significance | only slightly less than significant | very close to the established statistical significance level of p=0.05 |
| **88** | approached acceptable levels of statistical significance | equivocal significance | near significance | only slightly missed the conventional threshold of significance | very close to the threshold of significance |
| **89** | approached but did not quite achieve significance | essentially significant | near to statistical significance | only slightly missed the level of significance | very closely approaches the conventional significance level |
| **90** | approached but fell short of significance | extremely close to signiﬁcance | near significance | only slightly missed the significance level | very closely brushed the limit of statistical significance |
| **91** | approached conventional levels of significance | failed to reach significance on this occasion | near-borderline significance | only slightly non-signiﬁcant | very narrowly missed significance |
| **92** | approached near significance | failed to reach statistical significance | near-certain signiﬁcance | only slightly significant | very nearly significant |
| **93** | approached our criterion of significance | fairly close to significance | nearing significance | partial significance | very slightly non-significant |
| **94** | approached significant | fairly significant | nearly acceptable level of significance | partially significant | very slightly significant |
| **95** | approached the borderline of significance | falls just short of standard levels of statistical significance | nearly approaches statistical significance | partly significant | virtually significant |
| **96** | approached the level of signiﬁcance | fell just short of significance | nearly borderline significance | perceivable statistical significance | weak significance |
| **97** | approached trend levels of significance | fell barely short of significance | nearly negatively significant | possible significance | weakened significance |
| **98** | approached, but did reach, significance | fell just short of significance | nearly positively significant | possibly marginally significant | weakly non-significant |
| **99** | approaches but fails to achieve a customary level of statistical significance | fell just short of statistical significance | nearly reached a significant level | possibly significant | weakly significant |
| **100** | approaches statistical significance | fell just short of the traditional definition of statistical significance | nearly reaching the level of significance | possibly statistically significant | weakly statistically significant |
| **101** | approaching a level of significance | fell marginally short of significance | nearly significant | potentially significant | well-nigh significant |
