# Supplementary material for: Analysis of 567,758 randomized controlled trials published over 30 years reveals trends in phrases used to discuss results that do not reach statistical significance
Source: PLoS Biol. 2022 Feb 18;20(2):e3001562. doi: 10.1371/journal.pbio.3001562 (PMC8893613; doi:10.1371/journal.pbio.3001562)
Supplement: S2 Table — The colors represent the strength of evidence as specified in the main text. BF, Bayes factor. (DOCX) [file pbio.3001562.s004.docx]

**S2 Table**. The evidence of temporal change in the phrases with at least five time-points expressed as the Bayes factor relative to no temporal change (lower threshold set to 2.0). The colours represent the strength of evidence as specified in the main text.

| **Bayes factor** | **Phrase** |
| --- | --- |
| 9023.2 | "a positive trend" |
| 5970.0 | "a numerical trend" |
| 4381.4 | "an increasing trend" |
| 1990.3 | "nominally significant" |
| 1316.2 | "approaches statistical significance" |
| 363.9 | "difference was apparent" |
| 338.0 | "all but significant" |
| 198.3 | "not quite significant" |
| 158.5 | "failed to reach statistical significance" |
| 110.4 | "did not quite reach statistical significance" |
| 77.9 | "a decreasing trend" |
| 71.8 | "potentially significant" |
| 67.2 | "a slight trend" |
| 64.0 | "just failed to reach statistical significance" |
| 50.9 | "a definite trend" |
| 39.5 | "significant tendency" |
| 27.6 | "not yet significant" |
| 25.4 | "possible significance" |
| 24.0 | "a marginal trend" |
| 21.1 | "nearly significant" |
| 19.7 | "approaching significance" |
| 17.8 | "trending towards significance" |
| 17.4 | "uncertain significance" |
| 16.0 | "not conventionally significant" |
| 15.5 | "practically significant" |
| 15.0 | "marginally statistically significant" |
| 11.3 | "only slightly significant" |
| 10.0 | "a statistical trend" |
| 7.6 | "probably not significant" |
| 7.0 | "a negative trend" |
| 6.8 | "barely significant" |
| 5.8 | "an overall trend" |
| 5.4 | "slightly significant" |
| 4.9 | "a reliable trend" |
| 4.5 | "almost significant" |
| 4.2 | "tended to be significant" |
| 4.0 | "almost achieved significance" |
| 3.9 | "trend significance level" |
| 3.8 | "on the borderline of significance" |
| 3.7 | "almost insignificant" |
| 3.5 | "tendency toward significance" |
| 3.2 | "a favorable trend" |
| 3.1 | "not quite reach the level of significance" |
| 2.9 | "almost statistically significant" |
| 2.9 | "moderately significant" |
| 2.9 | "approached conventional levels of significance" |
| 2.8 | "not highly significant" |
| 2.8 | "slight significance" |
| 2.7 | "a notable trend" |
| 2.7 | "just failing to reach statistical significance" |
| 2.7 | "marginally nonsignificant" |
| 2.5 | "just failed significance" |
| 2.5 | "an observed trend" |
| 2.5 | "possibly significant" |
| 2.4 | "scarcely significant" |
| 2.3 | "at the margin of statistical significance" |
| 2.3 | "just failed to be significant" |
| 2.3 | "an unexplained trend" |
| 2.3 | "barely missed statistical significance" |
| 2.2 | "weak significance" |
| 2.2 | "modestly significant" |
| 2.0 | "fairly significant" |
| 2.0 | "not fully significant" |
| 2.0 | "likely to be significant" |
| 2.0 | "almost attained significance" |
| 2.0 | "a statistical trend toward significance" |
