# Supplementary material for: Analysis of 567,758 randomized controlled trials published over 30 years reveals trends in phrases used to discuss results that do not reach statistical significance
Source: PLoS Biol. 2022 Feb 18;20(2):e3001562. doi: 10.1371/journal.pbio.3001562 (PMC8893613; doi:10.1371/journal.pbio.3001562)
Supplement: S3 Table — (DOCX) [file pbio.3001562.s005.docx]

**S3 Table**. All extracted P values within the three range categories, as the proportion of the total of 11,926 extractions.

| **Category** | **K** | **proportion** | **95% confidence interval** |
| --- | --- | --- | --- |
| <0.05 | 2052 | 0.172 | 0.165-0.179 |
| >=0.05-0.15 | 8126 | 0.681 | 0.673-0.69 |
| >=0.15 | 1748 | 0.147 | 0.14-0.153 |
