# Supplementary material for: Analysis of 567,758 randomized controlled trials published over 30 years reveals trends in phrases used to discuss results that do not reach statistical significance
Source: PLoS Biol. 2022 Feb 18;20(2):e3001562. doi: 10.1371/journal.pbio.3001562 (PMC8893613; doi:10.1371/journal.pbio.3001562)
Supplement: Attachment — Submitted filename: Rebuttal_defOkt2021.docx [file pbio.3001562.s006.docx]

Dear reviewers,

Thank you for your very constructive comments. They have significantly improved the manuscript. We address them in a point-by-point fashion. We have numbered the comments per reviewer and made them bold. After the comment, we respond to the comment with an answer in plain text. Followed by the changes made in the manuscript, due to that specific comment. These changes are highlighted in blue.

Thanks!

Warm regards,

Joeri Tijdink, MD PhD

Amsterdam

Reviewer #1: [identifies herself as Agnes Dechartres]

***1. Review Plos Biology PBIOLOGY-D-21-01082R1, « Almost significant : trends and p values in the use of phrases describing marginally significant results in 567,758 randomized controlled trials published between 1990 and 2020 » This is a research on research report evaluating the prevalence of common phrases associated with non significant results in 567,758 randomized controlled trials (RCTs) published between 1990 and 2020, the trend over time and the associated p-values in a subset of RCTS. The topic is interesting and fits well the meta-research section of Plos Biology. The manuscript is overall well-written.
Please find below my comments. I hope you will find them useful.***

**Answer:** We thank the reviewer her useful insights. We address them one-by-one below and we think it has improved the manuscript substantially.

***2. Major comments :
-       The reference and link with spin should be made more apparent.***

**Answer:** We have added a description on misrepresentation (spin) in RCTs to the manuscript as this is indeed connected with our reporting strategy characterization.

We now write on page 4, L:23:

*‘Authors could misrepresent non-significant trial results through biased emphasis or phrasing. A well-known example of this so called ‘spin’ practice is switching the emphasis from non-significant primary to significant secondary outcomes. This highlighting favorable results while suppressing unfavorable data is considered as misrepresentation [5]. Another example is the use of linguistic spin. Linguistic spin could distort the interpretation of trial results in reframing or modifying the reader’s perception into a beneficial interpretation despite a statistically nonsignificant difference in the primary outcome [6].*

*Distracting reporting strategies are prevalent in trial reports, both in full-text and abstract reports. A selection of last year’s spin characterization indicates high percentages of detected spin across medical disciplines. For example, the detection of spin in 62.2% of 111 orthodontic RCTs [7], the detection of spin in sixteen abstract texts (55%) and eighteen main texts (62%) of 29 bariatric surgery RCTs [8], the detected spin in 35 of the 53 (66%) abstracts from orthopedic trauma RCTs in the top five orthopedic journals [9] and detected spin in 24 (47%) RCT articles in the top five of obstetrics and gynecology journals [10].’*

***3. -       We need more information on the methods section on how RCTs were identified (were they all those indexed as randomized controlled trials in publication type?), how did the authors exclude those that were not actual RCT reports?***

**Answer:** We have added the PubMed query to the manuscript in the methods section on page 7. We use the PubMed trial identification mesh-term and additional filter studies with the word ‘protocol’ in the title. The librarian’s assigned mesh-term may hypothetically contain misclassifications, but our impression is – based on the text inspections during the manual classification labor – that PubMed’s trial identification is sufficiently accurate for our study aim. Besides, we do not think that false negatives (trials not included) nor false positives (biomedical studies identified as trials) will have a significant effect as i) the number of included trials is large and ii) the statistical workflow in trial-like biomedical studies is often similar to trial reports.

We have now added the following text to the methods section (page 7, line 3):

*‘A flowchart shows the consecutive processing steps (Figure 1). We identified all RCTs in the PubMed database and excluded animal studies, non-English studies and studies that were not actual RCT reports [Sep 20, 2020] with the follow query: “All[SB] AND Humans[MESH] AND English[LANG] AND Clinical Trial[PTYP] NOT protocol[TITLE]”. Our custom search query is not previously validated. However, PubMed’s internal ‘Clinical trial’ filter is characterized with an average sensitivity of 87.3%, specificity of 34.8% and precision of 54.7% [17]. In comparison: A clinical query optimized for sensitivity reached 92.7% sensitivity and 16.1% specificity, whereas a clinical query optimized for specificity reached a sensitivity of 78.2% and a sensitivity of 52.0%. Our query is a thus a compromise between sensitivity and specificity. We expect that further restricting the query to ‘Humans’, ‘English’ and no protocols will have increased our specificity [17]. ‘*

**4.	How did the authors processed technically to search for the common phrases associated with non-significant results?**

Answer: We have clarified this in the method section on page 7, line 15. We now write:

*‘All trial PDFs were converted to XML and subsequently plain text. We converted the plain text to lower case and removed diacritical symbols as there are various types of quote Unicode characters. Subsequently we searched for exact matches (i.e., grep commandline tool) of the predefined phrases in this cleaned text. ‘*

**5.       The authors evaluated as a second step, p-values following the phrases commonly associated with a non-significant result in a subsample of 29,000 RCTs. Why 29,000? as they did that quite automatically, was it not possible to do it for the whole sample?**

Answer: This second step was not automatic, but demanded manual classification. Manual classification of the extracted sentences is very labor intensive and monotonous work. We considered a random selected subset to provide the same global patterns. We therefore distributed sets of 1,000 sentences to each of the authors to classify. Two authors initially labelled 4,000 sentences. Additional sets of 5,000 per author results in a total of 29,000. We have covered a substantial part of the full set with this sample and find clear distributional P value patterns. Therefore, we concluded that selection bias with this relative large subsample of 29,000 is unlikely.

We have clarified how we came up with the classification of 29,000 sentences and describe how we selected the sample size of the manual extraction. We now write in the manuscript in the methods section on page 9, line 7:

*‘We tried to quantify the first type of referral by manually extracting the P value within the first 100 characters directly following the extracted phrases within 29,000 random sampled phrases for the full set of phrases. This sample size was achieved through distributed labeling with all authors independently extracting P values from 5,000–7,000 sentences. We evaluated our interrater P value variability based on fifty sentences shown to two raters but mixed within the larger set of extractions and expressed as the mismatch percentage.’*

**6.       There is a contradiction in the Discussion section of the manuscript between what the authors did and showed : the prevalence of common phrases associated with non-significant results, mainly with a p-value between 0.05 and 0.15 is relatively low but still present (and this should probably be more highlighted and discussed, the prevalence seems low but it concerns all RCTs and many of them may be positive so one limitation is that they did not give the prevalence of such common phrases among RCTs with non-significant results) and seem steady over time. And the implications they highlight and the conclusion they reach: the abandon of formal statistical significance cutoff. The conclusion should be more in line with the results.**

Answer: Indeed. We have added this limitation to the limitation section in the discussion on page 15, line 13. We now write**:**

*‘Sixth, it remains unknown whether some trials may have had non-significant results and used different sentences to describe these results. This may have caused an underestimation of the prevalence of these types of sentences. ‘*

**7.       The authors mentioned in the discussion that it was previously discussed to lower the default p value threshold to 0.005. I think it was mainly proposed for pre-clinical research. Do the authors think it is applicable for clinical research including clinical trials?**

This is a good point. To our understanding, lowering the threshold has also been discussed for RCTs. For example: JAMA. 2018;320(17):1813-1815. doi:10.1001/jama.2018.12288 and corresponding discussion in JAMA. 2019;321(15):1532-1533. doi:10.1001/jama.2019.0566 and JAMA. 2019;321(15):1533. doi:10.1001/jama.2019.0574

A recent retrospective RCT investigation showed that shifting the threshold of statistical significance from P value < 0.05 to < 0.005 would have limited effects on medical intervention recommendations as eighty-five percent of recommended interventions showed P values below 0.005 for their primary outcome .

We have added this evidence in the discussion section of the manuscript. See page 17, line 5:

*‘Although the detrimental effects for medical intervention data are disputed [26-28], a recent retrospective RCT investigation showed that shifting the threshold of statistical significance from P value < 0.05 to < 0.005 would have limited effects on medical intervention recommendations as eighty-five percent of recommended interventions showed P values below 0.005 for their primary outcome [29].* *We are also aware that his will come with new problems and ways to game this new artificial statistical threshold.’*

**8. Minor comments:
-       Title: I suggest rephrasing the title as the current version is not completely clear something like:
« Almost significant » : Prevalence and trend of common phrases associated with non-significant results in 567,758 randomized controlled trials published between 1990 and 2020**

We have rephrased and clarified the title into: “*Almost significant: Prevalence and trends of common phrases associated with marginally significant results in 567,758 randomized controlled trials published between 1990 and 2020*”.

We prefer ‘marginally’ rather than non-significant as we investigated the full range of P values.

**-       Abstract: the authors state that « The question is how non-significant outcomes are reported in RCTs over the last thirty years. » They should be careful in wording as this is not what they evaluated here. They only evaluated the prevalence of common phrases associated with non-significant results in RCTs (and not specifically in non-significant ones). To answer the first question, they should have first identified a sample of RCTs with non-significant results and evaluated how they were reported**

Answer: We thank the reviewer for this important remark. We changed this aim in the abstract to: ‘*The question is how phrases expressing non-significant results are reported in RCTs over the past thirty years.’*

**-       Abstract: This should be made clear in the abstract that the authors extracted associated p-values in a subsample of RCTS as the sentence ending with « in 567,758 RCT full texts between 1990 and 2020 and manually extracted associated p values » may be misleading**

Answer: We have changed this in the abstract.

**-       Abstract: rephrase the sentence « Phrase data was modelled with Bayesian linear regression » to something like: temporal trend in phrase data was modelled with Bayesian linear regression**

Answer: Thank you for this suggestion. We have changed this in the abstract.

**-       Abstract : the conclusion should be more directly linked to the results**

Answer: We have changed the concluding text in the abstract. We now write:

*‘Our results demonstrate that phrases describing marginally non-significant results are regularly used in RCTs to report P values close to but above the dominant 0.05 cut-off**. The fact that the prevalence of the phrases remained stable over time indicates that this practice of rather broadly interpreting P values close to a predefined threshold remains prevalent.’*

**-       One of the phrases considered as over-interpretating non significant results is « failed to reach statistically significant results. » This phrase for me reflects the truth regarding achieving or not the significance threshold and I am not sure that it corresponds to an over-interpretation**

Answer: That is correct. The amount of spin varies between phrase. However, ‘failed to reach statistically significant results’ could also be phrased more neutral as: ‘non-significant results.’ The ‘reaching’ towards significant is in our opinion a little bit suspicious. There are more obvious examples, so we removed this one from the abstract and added this interpretation difficulty to the discussion. We now write in the discussion section on page 15, line 19:

*‘Finally, not all predetermined sentences actually represent a p value above 0.05. (e.g. ‘marginally significant’). However, in the manual analysis, we hardly found p values lower than 0.05 that corresponded with specific phrases.* *For example, the phrase ‘failed to reach statistically significant results’ is highlighting a fact, although not as neutral as simply stating: ‘non-significant results’. The amount of spin may therefore vary between phrases and potentially overreport some of our individual phrase prevalence estimations that describe marginally significant results’.*

**-       The introduction is a bit long and sometimes a bit pedantic. Some sentences are vague and not really clear. Among these, for example « Individual clinical researchers are subject to regulations, traditions and procedures… ».**

Answer: we have gone through the introduction section and deleted words and sentences and identified some words that may be interpreted as vague, and made these potentially vague sentences more concrete. We rewrote that specific sentence above and now write:

*‘Individual clinical researchers are subject to the mythical heritage or paradigm of the peculiar and well recognized 0.05 significance threshold (P= 0.05, alpha) that claims that findings below this predefined value reflect a true finding..’*

**-       In the introduction, the authors mention two previous meta-analytic studies showing that positive reporting and interpretation of primary outcomes in RCTs were frequently based on non-significant results. This sentence should be corrected as these studies were not meta-analyses. In addition, the sentence is misleading as these studies evaluated the prevalence of spin (misleading language) in RCTs with a non-significant primary outcome.**

Answer: We have corrected the description of the referred studies.

**-       Methods section : The sentence page 6, starting with « To increase the robustness of individual phrase prevalence estimations we binned… »**

Answer: We changed this sentence in the methods section on page 8, line 4 and now write:

*‘To increase the temporal robustness of individual phrase prevalence estimations, we binned RCTs according to their date of publication into periods of three years.’*

**Textual suggestions from reviewer 1:**

**-       A flow chart would be helpful**

We added a Flowchart (Figure 1 in current version) of the study processing pipeline.

**-       In the results section, the sentence page 10 starting with interrater p value variability… «  is not really clear. This was not announced in the methods.**

Answer: This is based on an internal validation to guarantee a similar scoring behavior among all co-authors. We have added this to the Methods section on page 9:

*‘We evaluated our interrater P value variability based on fifty sentences shown to two raters but mixed within the larger set of extractions and expressed as the mismatch percentage.’*

**-       Discussion section : sentence at the end of page 14 ending with « irrespective of the publication status ». I think this is rather the opposite : With that process, publication does not depend on study results**

Answer: We have changed the sentences in the discussion section on page 18, line 12 and now write:

*‘When the data are collected, the authors can finalize their paper with the results and interpretation, knowing that the paper is already accepted.’*

Reviewer #2:

**The manuscript reports on a meta-research study on how authors of reports on randomized controlled trials (RCTs) use specific wordings suggesting "marginally significant" results. The number of articles analyzed is impressive (grossly 570,000, more than 80% of all published RCTs that can be found in PubMed). But automatic analysis of such a large number also makes it difficult to attain a fine granularity, and this raises many issues. This is only imperfectly mitigated by manual extraction of P-values corresponding to a random sample of 29,000 extracted phrases. On the positive side, I must acknowledge that this, also, represents an enormous amount of work.**

Answer: Thank you for your kind words.

Major comments

**1.      Overall, the manuscript is unclear on what problem is precisely studied. Mostly the issue would be related to the use of threshold P-values for statistical testing. But actually, the issue is broader, and this is imperfectly tackled in the manuscript. The suggestion to lower the P-value threshold from 0.05 to 0.005, as proposed by John Ioannidis, is discussed in terms of lowering false positive reporting, but this is actually a completely different issue. Undoubtedly, authors would develop strategies to describe a P-value of 0.0051 as 'marginally significant', a 'trend to significance', etc. In a similar way, thresholds for Bayes factors are presented in the manuscript. It is mentioned that there is no evidence that Bayes factors would be reported with suspicious phrasing, but one may argue that Bayes factors there is no evidence the Bayes factors are reported in RCTs at all (despite this being advocated, references should be given in the discussion when coming to this).**

Answer: We have tried to better clarify what problem is actually studied in our manuscript at the end of the introduction section by highlighting the aims of the study. The main problem according to us with the use of these phrases is that authors would be incentivized to report statistically significant results only, with consequences such as P-hacking and p-fishing expeditions that authors report to come up with a significant test in order to get published.

We now write in the introduction section on page 6, line 12:

*‘Therefore, the study aims to detect the prevalence of specific non-significant phrases in RCTs and explore what types of phrases are used when reporting statistical non-significant findings to explore further the problems that arise with the significant threshold of p<0.05. We do this by…’*

We also clarify that the suggestion to lower the threshold would not prevent the p<0.05-problem in the discussion section may not be the best solution. Most likely it will only shift the problem to a lower reporting threshold and the significance dance then will be executed in the 0.005 range. We write in the discussion section on page 16, line 5:

*‘Although the detrimental effects for lowering the threshold for statistical significance for medical intervention data are disputed [26-28], A recent retrospective RCT investigation showed that shifting the threshold of statistical significance from P value < 0.05 to < 0.005 would have limited effects on medical intervention recommendations as eighty-five percent of recommended interventions showed P values below 0.005 for their primary outcome [29].**We are also aware that his will come with new problems and new ways to game this new artificial statistical threshold.’*

**Apart from an easy joke, if RCTs were routinely reported with Bayes factors and the thresholds given almost universally endorsed, then—again—authors may begin a 'significance dance' to advertise a Bayes factor of 3.18. The discussion also advocates the use of effect sizes and confidence intervals. But this is another issue, also. How many of the reports analyzed also reported measures of effect size and a confidence interval? Perhaps a vast majority of them, even those using suspicious phrasing. Moreover, a 95% confidence interval also somehow emphasizes a significance level at 0.05. Of course, it brings much more information. But how many readers will look whether the 95% CI of the relative risk includes 1 or not? All these issues are mixed up in the manuscript, and it would benefit from better delineating precisely the underlying problems. It seems to me that they are primarily caused by the use of thresholds for decision, whatever they are. But the authors may have a
different view. It should simply be clarified.**

We thank the reviewer for this important remark. We agree that the main problem is the use of an artificial threshold. We have adjusted the introduction section, as already highlighting the aims better and come back to this in the discussion section.

We now write in the discussion section on page 17, line 10:

*‘We are also aware that his will come with new problems and ways to game this new statistical artificial threshold of 0.005.’*

And page 17, line 21:

*‘Despite this improvement, we remain skeptical whether this will not shift the problem and stimulate researchers to overly report their effect sizes and confidence intervals.’*

**2.      As a follow-up comment, the topic may be narrowed to how authors phrase results close to, but above, the significance threshold, or enlarge it to envision what could be done to solve the issue (if it is really problematic). Currently, the manuscript is quite in-between. But the larger vision would raise many other issues: how do we dimension a study if there is no decision rule? (the power calculation would not apply) When should be stop a study without decision rules? Do we need a clear-cut conclusion of a RCT? (I would tend to say "no", but other authors have done sensible comments on that, and this relates to the suggestion to judge clinical relevance of RCTs on a case-by-case basis in the discussion; however on that specific point, I would tend to consider that this is still the case, whatever wording the authors use to describe their results). Bayes factors are used here, and are interesting. But perhaps a good option may be to analyze RCTs in a Bayesian framework,
and report posterior probabilities for decision making (again one may differentiate between decision-making for a particular trial, i.e. stopping recruitment or not, and decision-making about the drug or intervention evaluated). Other tools have also been proposed to improve the interpretation of RCTs (see e.g. Shakespeare Lancet 2001; 357: 1349-53).**

Answer: we thank the reviewer for this thoughtful comment as it raises some important clinical considerations that one need to take into account. To a certain extent, the question should always focus on the clinical relevance of an RCT. If the outcomes are clinically relevant is still a matter of debate that needs new argumentation.

We have addressed this issue in the discussion section.

We now write on page 18, line 20:

*‘We hope this study will stimulate researchers to put their creativity to good use in scientific research and abandon a narrow focus on fixed statistical thresholds but rather judge statistical differences in RCTs on its effect sizes and clinical merits.’*

**3.      Last on this broad issue, the topic of p-hacking could also have been covered, and there have been large-scale analyses of the distribution of P-values on that topic, too (e.g. Adda et al. PNAS 2020).**

Answer: We have added this suggestion to the reference list and refer to this article in the discussion section.

**4.      The end of the introduction suggests the need to study how results with P-values just above 0.05 are described in RCTs, but the last paragraph and the study does it the other way round (what are the P-values for predefined 'non-significance-related phrases'), and that way, the question looks less pertinent.**

Answer: We thank the reviewer for this insight. We have changed this and put more emphasis on spin in the introduction. For more details, see the answer to reviewer 1.

**5.      A huge work has been done to search a predetermined set of phrases reportedly associated with 'non-significant' results. But actually, there is no good evidence that those phrases indicated a 'significance dance' from the authors. Why not describe a result with P=0.048 (assuming a 0.05 predefined threshold) as 'marginally significant'? And actually, the figure 3 shows P-values as low as 0.01, and the figure 4 shows some sentences with a fair proportion of P<0.05. So all the phrases do not correspond to 'almost significant' results. I acknowledge that the peak at 0.05-0.10 is impressive, but the text should better reflect that all those statements did not correspond to P-values > 0.05.**

Answer: We agree with the reviewer that not all sentences are necessarily corresponding with non-significant results, as can be seen from Figure 3 – although in some cases the predefined threshold is lower than 0.05. (Cf. our mentioning in the Discussion section: “not all phrases are equally specific in their association with P values just above 0.05”. We now further highlight this issue in the discussion section. We now write on page 15, line 19:

*‘Finally, not all predetermined sentences actually represent a p value above 0.05. (e.g. ‘marginally significant’). However, in the manual analysis, we hardly found p values lower than 0.05.’*

**6.      I was surprised that only 272 of the 505 predefined phrases were encountered in half a million RCT reports, since those sentence were encountered in the literature. The blog apparently also accounts for the psychology literature and non-randomized studies, but this was striking. Also, I failed to access the URL given for that blog. It should therefore be checked for accurateness.**

Answer: The phrases not found were relatively long. The longer the phrases, the less likely to exactly match with the trial texts. The URL is indeed not working anymore. We have revised it and added new URL to the manuscript and put this address in the Github repository: <https://mchankins.wordpress.com/2013/04/21/still-not-significant-2/>

**7.      I wondered whether sentences such as 'did not quite reach statistical significance' actually correctly interpret the P-value as being above the significance threshold. I am not a native English speaker, so that I may miss the exact meaning of the sentence, but looking at such a sentence, I would understand 'P > 0.05' but perhaps not very high. This sounds different from 'marginally significant' or 'borderline significant' or 'almost significant'. On the sentences, also, some also seem to me to indicate statistical significance. For instance I would interpret 'nominally significant' as 'P<0.05, but close too'. If the study had extracted P-values between, say, 0.05 and 0.20 and than looked at how they were described, the story would be different. But it seems that all sentences searched were not equally suggesting a spin towards significance when P>0.05. This is discussed (second limitation), but too shortly.**

Answer: We agree with the reviewer and have added an extra limitation to the limitation section in the discussion. We now write:

*‘Sixth, it remains unknown whether some trials may have had non-significant results and used different sentences to describe these results. This may have caused an underestimation of the prevalence of these types of sentences.’*

**8.      The P-value threshold at 0.05 is most common. But some trials may have predefined other thresholds. At least for the RCTs for which P-values were manually extracted, did the authors look for such information?**

Answer: Unfortunately, in our automated search we only looked at p values with the 0.05 threshold in mind so we have no information on other thresholds. Additionally, this might (partially) explain why in Figure 3 p-values <0.05 are also found (although a minority). We tried to address this issue (see answer to point 5 above).

**9.      On what basis was it decided to manually extract P-values for  29,000 phrases?**

Answer: Since we also questioned whether the phrases actually corresponded with p values close to 0.05 and also questioned the automated search for reasons outlined in the discussion section, we decided that a manual analysis of a subset would eliminate our doubts and made the results and our analysis more rigorous. We now detail this in the methods section on page 9, line 7:

*‘We tried to quantify the first type of referral by manually extracting the P value within the first 100 characters directly following the extracted phrases within 29,000 random sampled phrases for the full set of phrases. This sample size was achieved through distributed labeling with all authors independently extracting P values from 5,000–7,000 sentences. We evaluated our interrater P value variability based on fifty sentences shown to two raters but mixed within the larger set of extractions and expressed as the mismatch percentage.’*

**10.     The very end of the abstract advocates the reporting of exact P-values. This is however not directly related to the study results. And actually, it seems that it was possible to extract the exact P-values corresponding to many extracted phrases. So, they were actually reported.**

Answer: Yes this is indeed correct. We have deleted the word *exact* from the abstract.
 **11.     Another important issue is how to interpret secondary outcomes of RCTs. Actually, many argue that, in the absence of correction for multiplicity or gatekeeping approach, one should not look at the P-values for secondary outcomes. Moreover, although this is unfortunately not always the case, the conclusion of a RCT on efficacy should target the primary outcome. It would therefore be interesting to distinguish primary and secondary outcomes here. I however understand that this would be virtually impossible to automatize.**

Answer: The reviewer is correct. Since we use automated software it is very hard to determine which p value correspond with which outcome. Besides, changing the outcomes of a trial based on the results of the study is a questionable research practices that is still quite prevalent (Haven et al 2019, Gopalakrishna et al 2021).

Additional comments

**1.      The term 'phrase model' is unclear.**

Answer: We removed this confusing term. We meant regression model and changed accordingly.

**2.      The introduction refers to 'low evidence results'. Yet, as the authors later acknowledge, the information brought by a study is not so different if it achieved P=0.049 or P=0.051. Moreover, many other elements may affect the level of evidence, or confidence in the evidence brought by the study, such as methodological characteristics, risk of bias, etc. This should be reflected in the sentence, or rephrased.**

Answer: Thank you for this remark. We have added some examples to this sentence. We now write on page 5, line 14:

*‘Insufficient evidence results (including factors such as high risk of bias, other methodological weaknesses, etc)’*

**3.      How was the density transformed to a percentage on the figure 3?**

Answer: We used a smoothed histogram with the bandwidth set to the standard deviation of the P values.

**4.      I wondered what 95% 'proportional' confidence intervals were (legend of figures 1 and 4). Are they the confidence interval of the proportion? Is that a common wording? (I never saw that).**

Answer: We used confidence interval estimated for proportions with the Yates’ continuity correction. (Brown LD, Cai TT, DasGupta A. Interval estimation for a binomial proportion (with Comments & Rejoinder). Statistical Science 2001; 16(2):101-133. doi:10.1214/ss/1009213286). Given the various ways to determine 95% CIs we clarified this in the methods and reduced the legend text to just ‘confidence interval’.

Reviewer #3:

The authors aimed to assess the prevalence of a specific set of phrases describing non-statistically significant results in articles reporting the findings of RCTs. They used exact matching to identify instances of this corpus across 567,758 articles. I have the following comments to improve the reporting and conclusions:

**1)      The authors cited the work of David Chavalarias and colleagues (JAMA 2016) but they did not compare their findings to theirs. In particular, the sample of Chavalarias et al included RCTs as indexed by Medline and they already quantified the evolution over time of the prevalence of p value <0.05 (thus of p values >= 0.05). They already found that the proportion of p values <0.05 was 91.4% in RCTS (completely consistent with the findings of the authors of ~9%) and they also found that this proportion remained constant over time. Chavalarias et al. also assessed the statements associated to p values in a subsample.**

Answer: We thank the reviewer for this suggestion. We now add this to the discussion section on page 17, line 17:

*‘Chavalarias et al (REF) describe in their paper results that are related to the reporting of pv values, effect sizes and CIg; in the vast majority (88%) of the included RCTs they found the reporting of a p value <0.05. They also highlight that in 2-3% of the analysed abstracts, they found the reporting of Confidence intervals and 22% of the abstracts described effect sizes.’*

**2)      The premise of the article is that describing RCT results as 'almost significant' (or 'marginally significant) can influence the perception of readers. The findings show that 'almost significant' is not frequent (only 0.14%). 'Marginally significant' was more prevalent (1.36%) but still much smaller than the prevalence of p values > 0.05 (about 9%). In addition, the use of these phrases seem to be stable over time (in particular the phrases do not appear among increasing or decreasing trends in Figure 4). The conclusions of the authors do not seem to be consistent with these findings.** 

Answer: We thank the reviewer for this comment. We highlight that these specific phrases should be interpreted with caution. However, in our manual analysis, these phrases corresponded mainly with non-significant P values. We add to the discussion section on page 15:

*‘Finally, not all predetermined sentences actually represent a p value above 0.05. (e.g. ‘marginally significant’). However, in the manual analysis, we hardly found p values lower than 0.05 that corresponded with specific phrases. For example, the phrase ‘failed to reach statistically significant results’ is highlighting a fact, although not as neutral as simply stating: ‘non-significant results’*

**3)      The Methods section does not provide any detail regarding the identification of RCTs. Did the authors use a filter to query PubMed? Which one? See 10.5195/jmla.2020.912 What is the sensitivity and specificity of the filter? How did the authors address the false positives?**

Answer: We have now added the precise search query. The query uses an internal PubMed filter to select clinical trials. This filter is a trade-off between sensitivity and specificity. We removed potential false positives with the ‘Human’, ‘English’ and no protocol AND extensions.

We have clarified the text:

*‘We identified all RCTs in the PubMed database and excluded animal studies, non-English studies and studies that were not actual RCT reports [Sep 20, 2020] with the follow query: “All[SB] AND Humans[MESH] AND English[LANG] AND Clinical Trial[PTYP] NOT protocol[TITLE]”. Our custom search query is not previously validated. However, PubMed’s internal ‘Clinical trial’ filter is characterized with an average sensitivity of 87.3%, specificity of 34.8% and precision of 54.7% [17]. In comparison: A clinical query optimized for sensitivity reached 92.7% sensitivity and 16.1% specificity, whereas a clinical query optimized for specificity reached a sensitivity of 78.2% and a sensitivity of 52.0%. Our query is a thus a compromise between sensitivity and specificity. We expect that further restricting the query to ‘Humans’, ‘English’ and no protocols will have increased our specificity [17].*

**4)      The authors could not retrieve 18.53% of identified articles. What was this proportion across publication years? Did it change? Were the authors less likely to retrieve full-text articles of older articles? What is the implication on their findings?**

Answer: We have plotted the yearly prevalences in Figure 1 and this indicates that the non-identified articles are approximately equally distributed as the prevalences are stable.

The absolute number of published trials is constantly increasing. Therefore, more trials are available for recent periods. This results in a slight increase in uncertainty for the prevalence’s in early periods. We do not expect an impact on modelling as the sample size is still large and binning of individual years mitigates this uncertainty.

**5)      The URL provided in reference 11 is not accessible. I find it very problematic. Is the list of phrases from Academia Obscura including all 505 phrases used by the authors? How was this list developed? Is this corpus derived specifically from RCTs? Is it based on articles spanning from 1990 to 2020? In addition, how can the reader understand the extent to which these phrases are sensitive and specific to the reporting of non-significant results? The authors refer to "potentially associated with reporting non-significant results" but the reader has no way to make a judgment about the meaning of "potentially".**

Answer: We are sorry to notice that the link is offline. It is still available on web.archive.org [https://web.archive.org/web/20190821054730/http://www.academiaobscura.com/still-not-significant/]. The copy of the post is available here: https://mchankins.wordpress.com/2013/04/21/still-not-significant-2/ We have also added the list to GitHub: https://github.com/wmotte/almost_significant/blob/main/doc/still_not_significant.txt

**6)      The Methods section does not mention that the authors selected articles published in English. The proportion of excluded articles based on language is not reported.**

Answer: We had indeed not clarified our query and have changed that accordingly. We only selected trials written in English. We have not identified non-English papers as our phrases are only in English. Our results are therefore not generalizable to other languages. We have added this as a limitation to the Discussion on page 15:

*‘Third, we studied English-language RCTs only. Generalizations to other languages can therefore not be made.’*

**7)      233 (46%) phrases were not identified among the 567,758 pdf. What were the main differences between the 272 phrases found at least once and the 233 phrases?**

Answer: The phrases not found were relatively long. The longer the phrases, the less likely to exactly match with the trial texts.

**8)      The authors searched for exact matches. They should justify why they did not use approximate string matching methods? What is the implication on their findings?**

Answer: We did not implement an elastic search strategy, as this could potentially change the interpretation, for example, by removing the negation. With exact string matching we are certain that a specific spin-like phrase is written in the trial report. However, this may have led to underreporting and the actual prevalence of these phrases may be an underestimation. We highlight this in the discussion section as a limitation. See page 15, line 15.

*‘Seventh, we did not implement an elastic search strategy in our pdfs, as this could potentially change the interpretation, for example, by removing the negation. With exact string matching we are certain that a specific spin-like phrase is written in the trial report. However, this may have led to underreporting and the actual prevalence of these phrases may be an underestimation.’*

**9)      The authors grouped selected articles into 3-year periods. The first period would be 1990-1992 and the last period would be 2017-2019. How did they handle the last period from Jan to Sep 2020?**

Answer: We kept 2020 as an (incomplete) last period. We have added this to the methodology in the methods section.

**10)     There is no detail regarding the method for sampling articles to manually extract p values. Was random sampling stratified according to publication year or other factors?**

Answer: We randomly sampled from the full set of phrases.

**11)     Did the authors searched for exact matches in both the abstract and the main body of the selected articles? Were results different when stratified by location (abstract vs main text)?**

Answer: We searched for exact matches in both the abstract and full text and we made no distinction by location.

**12)     Figure 4 shows large percentages of p values <0.05 for certain phrases. Across all extracted p values, what is the frequency and percentage of p values <0.05? What is the explanation? Is it that the significant threshold was lower than 0.05 in these papers? Was it that the selected phrases are not specific enough? It seems to be a considerable limitation in face of the objective of the authors to assess reporting of non-statistically significant findings.**

Answer: We thank the reviewer for this important suggestion. Indeed this is a limitation that we tried to address in the limitation section by adding this in the discussion section (page 15, line 19 and our response to the other reviewers). Some phrases may not correspond with statistically non significant findings. However, in our manual analysis, we hardly found p values lower than 0.05 that corresponded with specific phrases.

**13)     In the Discussion, the authors conclude: "We present a robust estimate of nine percent of RCTs using specific language to report P values around 0.06." I suggest rephrasing. Because of the lack of information regarding the Methods and the limitations mentioned above, I find the claim that the estimate is robust undue. And I am not following 'p values around 0.06'.**

Answer: We agree with the reviewer. We now write in the discussion section on page 14:

*‘We present an estimate of nine percent of RCTs using specific phrases to report P values above 0.05.’*

**14)     In the abstract, the authors state "the phrase prevalence remained stable over time, despite all efforts to change the focus from P < 0.05 to reporting effect sizes and corresponding confidence intervals" Did they assess if effect sizes and confidence intervals were reported alongside non statistically significant results? If not, I suggest modifying this conclusion.**

Answer: Here we also agree with the reviewer and we have removed this specific sentence. We also allude to confidence intervals and effect sizes when we discuss the results in light of the outcomes of the Chavalarias et al manuscript in JAMA from 2016 in the discussion section. See above in this rebuttal letter as a response to you (reviewer 3, first point).
