# Supplementary material for: Analysis of 567,758 randomized controlled trials published over 30 years reveals trends in phrases used to discuss results that do not reach statistical significance
Source: PLoS Biol. 2022 Feb 18;20(2):e3001562. doi: 10.1371/journal.pbio.3001562 (PMC8893613; doi:10.1371/journal.pbio.3001562)
Supplement: Attachment — Submitted filename: RebuttalJan2022_PLoSBiology_def2.docx [file pbio.3001562.s007.docx]

Dear editors and reviewers,

Thank you for your constructive comments. They have significantly improved the manuscript. We address them in a point-by-point fashion. We have numbered the comments per reviewer and made them bold. After the comment, we respond to the comment with an answer in plain text. Followed by the changes made in the manuscript, due to that specific comment. These changes are highlighted in blue.

Thank you in advance.

Warm regards,

Joeri Tijdink, MD PhD

Amsterdam

Editorial comments:

**a) Please can you change your Title to "Analysis of 567,758 randomized controlled trials published over 30 years reveals trends in phrases used to discuss results that are not statistically significant" or "...that do not reach statistical significance" or "...that are only marginally significant," whichever is most accurate.**

Answer: We have changed the title of the manuscript accordingly to: *"Analysis of 567,758 randomized controlled trials published over thirty years reveals trends in phrases used to discuss results that do not reach statistical significance".*

**b) Please address the remaining concerns raised by the reviewers. While reviewer #3 remains critical, after discussion with the Academic Editor, we think that their concerns can be addressed by clarifying methods, toning down claims and making the limitations more explicit.**

Answer: See our response to the points raised by reviewer #3 below. We have improved the clarification of the methods, deleted some of the sentences that made claims and added limitations to the discussion section.

**c) Please could you supply a blurb, according to the instructions in the submission form? Write a short, appealing statement summarizing your research, about 20-30 words or 1-2 sentences long. If your submission is accepted for publication we'll include your blurb in emails to our readers, and on the journal homepage. Your blurb should, without exaggeration, entice people to read your manuscript.**

Answer: we suggest the following blurb: Researchers are using appealing phrases to describe non-significant findings as if they were below the p=0.05 significance threshold.

**d) Please check that you adhere to our guidelines pertaining to systematic reviews and meta-analyses (e.g. by including a completed PRISMA checklist and flow** diagram) [https://journals.plos.org/plosbiology/s/best-practices-in-research-reporting#loc-reporting-guidelines-for-specific-study-types](https://journals.plos.org/plosbiology/s/best-practices-in-research-reporting#loc-reporting-guidelines-for-specific-study-types)

Answer: No standard guidelines apply to our meta-research study (nor the systematic review PRISMA checklist) since it not a systematic review.

**e) Your current financial declaration says “The author(s) received no specific funding for this work.” Please can you confirm that this is correct.**

Answer: Hereby we confirm that we did not receive any funding for this work.

**f) Please address my Data Policy requests below; specifically, while we recognise that your raw data and code are presented in your GitHub deposition, we need you to supply the numerical values underlying Figs 2ABC,3, 4, 5, S1, S2. Please cite the location of the data clearly in each relevant Fig legend.**

Answer: We have added the link to the GitHub deposition in the legends of all the figures (2ABC, 3, 4, 5, S1, S2) and tables, so the reader can find all relevant data.

**As you address these items, please take this last chance to review your reference list to ensure that it is complete and correct. If you have cited papers that have been retracted, please include the rationale for doing so in the manuscript text, or remove these references and replace them with relevant current references. Any changes to the reference list should be mentioned in the cover letter that accompanies your revised manuscript.**

Answer: We have reviewed the references, and found no errors so we did not make any changes.

**Many thanks for providing the raw data and code in Github. However, we also need the individual numerical values that underlie the Figures of your paper be made available in one of the following forms:**

**1) Supplementary files (e.g., excel). Please ensure that all data files are uploaded as 'Supporting Information' and are invariably referred to (in the manuscript, figure legends, and the Description field when uploading your files) using the following format verbatim: S1 Data, S2 Data, etc. Multiple panels of a single or even several figures can be included as multiple sheets in one excel file that is saved using exactly the following convention: S1_Data.xlsx (using an underscore).**

Answer: We have used the PLoS Biology format to describe the supplementary files. However, since it is easier to share the data online, we have shared the link to the GitHub disposition. There readers can find all data that are related to the figures and tables: <https://github.com/wmotte/almost_significant/tree/main/Fig_and_Data>

**2) Deposition in a publicly available repository. Please also provide the accession code or a reviewer link so that we may view your data before publication.**

Answer: Indeed, the accession code was not delivered. Since the OSF is a bit more complicated to share data, we have put all the data (when possible) in a publicly available repository on GitHub. You can access these data with the following link: <https://github.com/wmotte/almost_significant/tree/main/Fig_and_Data>

**3) Regardless of the method selected, please ensure that you provide the individual numerical values that underlie the summary data displayed in the following figure panels as they are essential for readers to assess your analysis and to reproduce it: Figs 2ABC,3, 4, 5, S1, S2. NOTE: the numerical data provided should include all replicates AND the way in which the plotted mean and errors were derived (it should not present only the mean/average values).
IMPORTANT: Please also ensure that figure legends in your manuscript include information on where the underlying data can be found, and ensure your supplemental data file/s has a legend.**

Answer: We have linked the correct datasets to the corresponding figures and tables.

**Please ensure that your Data Statement in the submission system accurately describes where your data can be found.**

Answer: We have written in the data availability statement:

**Data sharing and availability**: All used PubMed IDs, detected phrases, co-text extractions, manually identified P values, and processing scripts are openly shared here: <https://github.com/wmotte/almost_significant/tree/main/Fig_and_Data>; and here: https://github.com/wmotte/almost_significant (v1.0; <http://doi.org/10.5281/zenodo.4313162>)

Reviewer #1:

Review PBIOLOGY-D-21-01082R2 manuscript entitled "Almost significant: Prevalence and trends of common phrases associated with marginally significant results in 567,758 randomized controlled trials published between 1990 and 2020"

**I would like to thank the authors for having answered my comments and modified their manuscript accordingly.
I have some last comments:

1.	Abstract section: it is not clear what represents the rate 68.1% at the end of results presented. Is it the % of p-values between 0.05 and 0.15? Please clarify**

Answer: Thank you for your suggestions. This is indeed unclear. We have changed that sentence in the abstract. We now write on page 3, line 2:

In a random sampled subset of 29,000 phrases the manually identified and corresponding 11,926 P values, 68,1% ranged between 0.05 and 0.15 (CI: 67.3–69.0; median 0.06).

**2.	Abstract section: in the conclusion, "demonstrate" is too strong. Please consider using "show" instead**

Answer: Thank you. We have used the word ‘*show’* now in the abstract.

**3.	The introduction is still very very long and I still find that some sentences or wording are too vague or unclear. For example: "The use of this fixed threshold has important disadvantages as the context determines the corresponding threshold". I am not sure that the catch-22 situation expression is clear for most researchers. Also not really clear "insufficient evidence results (including factors such as high risk of bias, other methodological weaknesses", "given the relatively rules-oriented RCT research environment", "the success of an RCT"**

Answer: We thank the reviewer for these points. We have made adjustments in the introduction section by deleting sentences (e.g. about the catch-22 situation). Furthermore, we have reduced the extensive description of the spin findings that were added in the first revision.

**4.	I am happy with the reference to spin in the introduction but I think it is not necessary to provide a catalog of findings from recent studies on that topic**

Answer: We have reduced the paragraph on spin and deleted the description of the examples on page 5 and summarized these examples in 1 sentence. We now write (page 5, line 4-5:

And finally, recent reports indicate a high percentages (ranging from 47-66%) of detected spin across medical disciplines. (7,8,9,10)

**5.	I also find that the plan of the introduction is not easy to follow. I really suggest to condense and to be more factual following a clear-cut plan**

Answer: By reducing the text of the introduction and have a clear line of reasoning in mind, we have made some changes, deletions and summarizing additions. We think we have improved the introduction and think it is now easier to follow. See manuscript with tracked changes on page 4,5 and 6.

**6.	I would be more careful in the formulation of the objectives in particular the part "explore what types of phrases are used when reporting statistical non-significant findings". It is not exactly what the authors did. This formulation suggests that they searched first for non-statistically significant results and then look at the sentences around. In this study, they did the contrary: they look the p-values following a sentence that could be related to a marginally significant result.**

Answer: Thank you for this remark. We have changed this objective and we now write (see page 6, Line 10-12):

`…determine what phrases correspond with the reported statistical non-significant findings to explore potential consequences that arise with the significant threshold of p<0.05.`

**7.	I think this is also a limitation to acknowledge more clearly: having focused on all RCTs and not only on those with non-statistically significant results**

Answer: We do not fully understand this limitation, as we think that obtaining an, as large as possible set of full-text RCT corpus, prevents biases. However, despite our sample size, we have missed other RCTs, obviously the non-English ones. This may have caused selection bias. This is mentioned in the Discussion with limitation 3 and 4 on page 16:

“Third, we studied English-language RCTs only. Generalizations to other languages can therefore not be made.” and 4: “Fourth, we only had access to published full texts.”

**8.	In the methods section, why did the authors use "clinical trial" as publication type and not "randomized controlled trial"?**

Answer: Pubmed’s terminology is somewhat misleading, given that both filters contain the selection of RCTs (i.e., , random*[Title/Abstract] OR random allocation[MeSH Terms] OR therapeutic use[MeSH] in the “sensitive/broad” filter). PubMed offers two RCT types of Clinical Study filters: a “specific/narrow” ‘randomized controlled trial’ version with a self-reported sensitivity/specificity of 93%/97% and a “sensitive/broad” version with a sensitivity/specificity of 99%/70% [https://pubmed.ncbi.nlm.nih.gov/help/#clinical-study-categories-bibliography]. Given the general profiling rather we valued the high sensitivity above the specificity. Also given the much lower number of RCTs which are selected if the “specific/narrow” filter is used. We have now added to the description of our query on page 7, line 9: “It is optimized for a sensitive and broad rather than a specific and narrow yield.”

**9.	Typo in the paragraph statistical analysis: principled**

Answer: we have removed the word principled on page 8.

**10.	Another sentence vague and pedantic in the methods: "with the tendency of humans to understand the world by applying thresholds to continuous spectra"**

Answer: we have removed this sentence from the manuscript as it has no added value to the manuscript.

**11.	In the results: the sentence "the overall phrase-positive RCT prevalence was stable over time (8.65%). Does it correspond as indicated to the overall prevalence, ie 49,134/567,758? It looks so but "stable over time" in the same sentence is confusing. I would make two sentences.**

Answer: Thank you for this detailed suggestion. We have adjusted the sentence and we now write on page 10:

The overall phrase-positive RCT prevalence was 8.65% (95% confidence interval: 8.58–8.73%) and this percentage was stable over time.

**12.	Results, associated p-values: I would expect here the prevalence of p-values in the range 0.05-0.15 and not only the median. Please also make two sentences**

Answer: we have made two sentences and we now write on page 12:

The P value distribution was characterized with a high prevalence within the 0.05–0.15 range with a median of 0.06. in the distribution of all detected P values, we found in the 25–75% interval P values between 0.05–0.08. The 5–95% interval had P values between 0.006–0.15. (See **Fig 4**).

**13.	Results, associated p-values: The sentence "the highest percentages for relative frequent phrases were found in this particular range.. " is unclear**

Answer: We have also adjusted this sentence and we think it is now clearer. We write on page 13:

The highest percentages of the following frequent phrases were found in this particular range of 0.05 -0.15 for:…

**14.	Discussion, in principal findings, "no specific reasons were found for these differences over time". For me, it is not really a finding as they did not report to study that. I would eventually discuss that point later in the discussion**

Answer: we have removed this sentence as it does not significantly add to the discussion at this point.
 **15.	The conclusion should be more in line with the results. In particular, the sentence "fifteen years of advocacy to shift away from null hypothesis testing has not yet fully materialized in RCT publications"**

Answer: Thank you. We have removed this sentence and changed the final paragraph slightly. See page 18.

**16.	The flow chart could be improved**

Answer: we have adjusted the flow chart and also included the RCTs that were excluded due to missing full texts. See Fig 1.

**17.	Figure 5: I would add Q1-Q3 with the median if possible**

Answer: we have added the range and the median to fig 5.

Reviewer #2:

I thank the authors for their answers and revision of the manuscript.

Some issues may still be considered:

**1.      When describing the "mythical paradigm" of statistical testing in the introduction, authors may be clear that the statistical framework does not mandate that P-values < 0.05 indicate a true effect and those >0.05 indicate no effect. This is simply a rule to reject the null or not, based on long-run properties allowing to control the type I and II (provided studies are correctly powered) error rates. So it is more the interpretation that has pervasively permeated the medical community that has led to the shortcut P<0.05 = true effect and P>0.05 = no effect. This may be clarified.**

Answer: This indeed needs to be clarified. We now have added to the introduction (P4, Line 11-13):

`However, the 0.05 threshold is a simple rule to reject the null hypothesis, controlling for type I and II errors. This has led to misinterpretations that dichotomized the P value (P <0.05 = true effect, P>0.05 = no effect)`

**2.      The flow chart should have arrows going out with the "excluded" studies at each step, and the reason why they would go out of the flow. For instance, when starting from the 696,842, 129,084 are excluded before full test download: they should appear on the chart, with the reason why full text was not retrieved (there could be several reasons for different sets of studies).**

Answer: We have updated the flow chart. The reason is a single one: no full-text available within our institutional subscription licenses. See Fig 1.

**3.      The sixth limitation is quite vague, and should be made more precise. I believe two reviewers alluded to this. The way the data were analyzed did not look at whether the RCTs achieved their primary outcome or not, nor if the sentences and p-values extracted related to the primary outcome. So the limitation should be clarified: we do not know if what is reported relates to the primary outcome or not, and there is no stratification according to whether the trials reached their primary endpoint (showed a "significant result" of their primary outcome) or not.**

Answer: Thank you for this suggestion. We were not sure whether this limitation was the sixth limitation addressed. However, we found this limitation important and we have added this one to the discussion section (P:15, L:19-21):

“Seventh, we do not know whether the P value and the corresponding significance phrase actually referred to the study´s primary outcomes or whether it described less important secondary or tertiary outcomes.”

Some minor points

**1.      Abstract, "RCTs regularly use specific phrases": RCTs themselves do not use any sort of sentence. Those phrases can be found in their reports. I would therefore write it differently, such as "Our results show that RCTs reports regularly contain specific phrases describing …" (not sure either there would be a need for a real demonstration).**

Answer: Indeed. We have changed the text accordingly.

**2.      In the introduction, I would not allude to the exact number of full-texts analyzed, since this is already part of results, but more on the strategy: "We do this by quantitatively analysing 567,758 RCT full-texts …" would be changed to "We do this by quantitatively analysing RCT full-texts in English registered in the last decade …"**

Answer: We have followed the reviewers advice and deleted the number of trials included.

**3.      What is the need for "direct" in the sentence "To obtain direct evidence on phrase changes …"? Would another type of model produce indirect evidence? Moreover, the evidence itself would be more in the data than in the model.**

Answer: Thank you for this sharp comment. We have deleted direct from the text in the methods section.

**4.      The figure 1 should be referenced in the text, e.g. "We obtained the full text of 567,758 full-texts of the total of 696,842 PubMed-registered RCTs (81.47%) (fig 1)".**

Answer: We have changed this in the text.

**5.      The seventh limitation (related to not using elastic search) could be put earlier, just after the fact that a fixed set of phrases was used: such a fixed set was used, and exact match was searched.**

Answer: Correct. We have merged these two limitations in the discussion section and started the limitation section with this point.


Reviewer #3:

**The response of the authors and the revised manuscript reveal flaws in the methodology. The methodology chosen by the authors does not allow answering the objective of assessing "how non-significant outcomes are reported in RCTs over the last thirty years" or "how phrases expressing non-significant results are reported in RCTs over the past thirty years". It is impossible to assess the robustness of the approach, but Figure 4 clearly shows that the approach is biased. In addition, these shortcomings are not acknowledged or are downplayed by the authors.**

Answer: We thank the reviewer for his or her feedback and we try to acknowledge the shortcomings in more detail. See below in this rebuttal letter the answer to the points by reviewer #3 raised.

**1)      The whole methodology relies on a list of 505 phrases compiled and shared through a blog post by Matthew Hankins. The manuscript does not explain how the list was developed. The original blog post only mentions: "The following list is culled from peer-reviewed journal articles in which (a) the authors set themselves the threshold of 0.05 for significance, (b) failed to achieve that threshold value for p and (c) described it in such a way as to make it seem more interesting."  So, the internal and external validity of the findings relies completely on a list of phrases developed by a single researcher based on an irreproducible non-systematic review of an ad hoc selection of articles of unspecified nature (biomedical research? RCTs?). As highlighted by all reviewers of this manuscript, the correct method should be to first identified a sample of RCTs with non-significant results and to evaluate how they are reported**

Answer: we agree with the reviewer that this list is not externally validated. However, we highlight that these phrases are potentially associated with non-significant results and we use them to test them in a real-life sample (namely 567,758 RCTs). One of the outcomes of our study is that we tested these 505 phrases (and came to the conclusion that only a minority is actually used regularly in RCTs). Furthermore, we highlight this as first limitation in the discussion section. See page 15.

**2)      The authors downplay this major flaw. They state "First, we [Matthew Hankins?] pre-defined more than five hundred phrases denoting results that do not reach formal statistical significance. We may have missed phrases with similar meanings. This would lead to an underestimated overall prevalence." Figure 4 shows that a certain proportion of p values associated with the phrases are below 0.05. It clearly invalidates the approach. It is also incorrect to state that the prevalence is underestimated. The authors had the opportunity to report the exact proportion of p values < 0.05 but they did not. It is difficult to guess the proportion based on Figure 4, a histogram would be better. It is the area under the pdf to the left of 0.05. The proportion of p values <0.05 seems to be at least 2.5%. It is a relatively large percentage compared to the estimated 9%. The authors minimize this finding by stating "Finally, not all predetermined sentences actually represent a p value above 0.05. (e.g. 'marginally significant'). However, in the manual analysis, we hardly found p values lower than 0.05 that corresponded with specific phrases".**

Answer: We thank the reviewer for this comment. Obviously, there is a subset of P values below 0.05. This is clearly visible in Fig 5 histograms, where the gray histogram bars represent this proportions for the individual phrases. Our interest is not so much with the absolute numbers related to this small <0.05 subset, but the large subset of P values >0.05 in *most* phrases.

We tried to be more specific about this potential flaw and we now write in the discussion section:

"Finally, not all predetermined sentences actually represent a p value above
0.05. (e.g. 'marginally significant'). However, we hardly found P values lower than 0.05 that corresponding with specific phrases in the manual analysis (see Table S3)".

**3)      The conclusion of the article "too much focus on formal statistical significance cut-offs hinders full transparency and increases the risk for misinterpretation and selective publication, particularly when P values approach but do not cross the 0.05 threshold." and of the abstract "To enhance responsible and transparent interpretation of RCT results, researchers, clinicians, reviewers, and editors should abandon the focus on formal statistical significance thresholds and stimulate reporting of P values with corresponding effect sizes and confidence intervals and focus on the clinical relevance of the statistical difference found in RCTs." are not supported by the findings and go well beyond them.**

Answer: We thank the reviewer for this remark. We have changed these sentences in the abstract and discussion section. We now write:

In the abstract: To enhance responsible and transparent interpretation of RCT results, researchers, clinicians, reviewers, and editors may reduce the focus on formal statistical significance thresholds and stimulate reporting of P values with corresponding effect sizes and confidence intervals and focus on the clinical relevance of the statistical difference found in RCTs.

In the discussion section: “too much focus on formal statistical significance cut-offs hinders responsible interpretation of RCT-results. It may increase the risk for misinterpretation and selective publication, particularly when P values approach but do not cross the 0.05 threshold.”
